# Evolution of Stereotactic Ablative Radiotherapy in Lung Cancer and Birmingham’s (UK) Experience

**DOI:** 10.3390/medicines5030077

**Published:** 2018-07-23

**Authors:** Sundus Yahya, Qamar Ghafoor, Robert Stevenson, Steven Watkins, Beshar Allos

**Affiliations:** Hall-Edwards Radiotherapy Research Group, Queen Elizabeth Hospital, Birmingham B15 2TH, UK; Qamar.Ghafoor@uhb.nhs.uk (Q.G.); Robert.Stevenson@uhb.nhs.uk (R.S.); Steven.Watkins@uhb.nhs.uk (S.W.); Beshar.Allos@uhb.nhs.uk (B.A.)

**Keywords:** lung cancer, radiotherapy, stereotactic ablative radiotherapy (SABR), stereotactic body radiotherapy (SBRT), radiofrequency ablation (RFA), microwave ablation, dosimetry, radiotherapy treatment techniques

## Abstract

Stereotactic ablative radiotherapy (SABR) has taken a pivotal role in early lung cancer management particularly in the medically inoperable patients. Retrospective studies have shown this to be well tolerated with comparable results to surgery and no significant increase in toxicity. Paucity of randomized evidence has dictated initiation of several trials to provide good quality evidence to steer future practice. This review summaries salient developments in lung SABR, comparisons to surgery and other platforms and our local experience at University Hospitals Birmingham, UK of lung SABR since its initiation in June 2013.

## 1. Introduction

Stereotactic ablative radiotherapy (SABR) refers to high precision radiotherapy with the aim of delivering an ablative dose of radiotherapy in fewer fractions with minimal dose to surrounding normal tissue. In lung cancer, SABR has been a major advancement in the treatment of early stage disease over the last decade. With more radiotherapy centres now providing this treatment, the role of SABR has evolved, particularly in patients who are medically inoperable or even when tissue biopsy is deemed high risk.

There remains paucity of randomised evidence for SABR and most data is based on retrospective case series. Multiple trials are currently being undertaken to answer various questions (including direct comparison with surgery in operable lung cancer) regarding the role of lung SABR. Owing to lower morbidity as compared to surgery in early stage non-small cell lung cancer (NSCLC), SABR has been widely adopted [1] particularly in frail patients with poor lung function reserve.

## 2. Comparisons of SABR vs. Surgery

There is no randomised data available at present comparing SABR versus Surgery in Early Lung Cancer. Chen et al. [2] published a meta- analysis attempting propensity score studies. They analysed sixteen studies showing similar lung cancer specific survival although overall survival favoured surgery (Hazard ratio [HR] for SABR vs. surgery, 1.48 [confidence interval, 1.26–1.72]; I2 = 80.5%).

Another study [3] reported on a pooled analysis of two randomised trials (STARS and ROSEL) both of which closed early due to slow accrual. These compared SABR vs. lobectomy for stage 1 NSCLC with SABR shown to be better tolerated and may confer better survival.

Several studies have reported comparisons of outcomes of SABR in inoperable and operable stage 1 lung cancer with favourable toxicity profile although further prospective randomised data is awaited.

Lagerwaard et al. [4] published outcomes after SABR in patients with potentially operable stage I NSCLC. This prospective study reported on 25% of lung SABR cases that were potentially operable. The median overall survival (OS) was 61.5 months at median follow-up of 31.5 months, with 1-year and 3-year survival rates of 94.7% and 84.7%, respectively. Regional and distant failure rates at 3 years were each 9.7%. They reported grade ≥3 radiation pneumonitis and rib fractures in 2% and 3%, respectively.

Similarly, a Japanese prospective study [5] compared 100 inoperable patients vs. 64 operable patients treated with SABR. Grade 3 and 4 toxicities were observed in 0.1% and 0.02% patients, respectively in the inoperable group with no grade 5 toxicity observed. In the operable group, 3-year OS was 76.5% (95% CI 64.0–85.1%) as compared to 59.9% (95% CI 49.6–68.8%) in the inoperable group. Grade 3 toxicities were 0.07% with no grade 4 and 5 toxicities observed in the operable group.

Sun et al. [6] has published 7-year follow up data for stage 1 non-small cell lung cancer treated with SABR. They reported estimated 5-year and 7-year progression-free survival rates of 49.5% and 38.2%, respectively; the corresponding overall survival rates were 55.7% and 47.5%, respectively. No. grade 4 or 5 adverse events were reported with only 4.6% grade 3 toxicity.

Our local survival data is comparable with most published series, with similar toxicity and control rates [7,8].

## 3. SABR vs. Other Treatment Modalities (Radiofrequency Ablation/Microwave Thermal Ablation MTA)

SABR has also been compared to other treatment modalities including radiofrequency ablation (RFA) and conventional radiotherapy and was found to be superior. A meta-analysis [9] reported similar overall survival at 1 year (68.2–95% vs. 81–85.7%) and 3 years (36–87.5% vs. 42.7–56%) between patients treated with RFA and SABR. However, 5-year survival was higher for SABR (47%) than for RFA (20.1–27%). The most common complication of RFA was reported to be pneumothorax (19.1–63%).

Although tumours of up to 5 cm can be treated with RFA, treating tumours ≤3 cm has been shown to confer superior control rates. There are other limitations to RFA including tumour location (apical, posterior, and/or central or closer to the scapula) due to difficulty in the application of electrodes.

A review published by Klapper et al. [10] explored alternatives to lobectomy for NSCLC including radiofrequency ablation (RFA) with variable local control rates (25–88%) and OS reported between 25% and 80% with RFA.

Other modalities include microwave thermal ablation (MTA). There is limited clinical data on the effectiveness of MTA owing to short follow-up periods and small patient numbers. However, MTA appears to be most effective in tumours sized less than 3 cm, similar to RFA. Survival data remains sparse, and only one study reported cancer-specific survival to be 61% at 3 years. Complications of MTA include pneumothorax; reported at 39–63%, with 8–16% of patients requiring a chest drain [10]. Table 1 shows comparison of thermal ablation (RFA and MTA) vs. SABR detailing the technical aspect, advantages/disadvantages and reported side effects bearing in mind the paucity of survival data for ablative techniques.

The LUMIRA trial [11] evaluated the effectiveness of radiofrequency (RFA) and microwave ablation (MWA) in lung tumours. The control rates and overall survival was comparable between the two groups testing RFA and MTA but owing to small sample size of 52, it is difficult to draw any conclusions.

## 4. Biologically Effective Dose (BED)

In accordance with current UK standards, UHB employs a 3, 5 or 8-fraction alternate-day SABR treatment schedule [14].

Biologically effective dose (BED) allows a physical dose to be translated into a meaningful biological effect it has on tumour growth as well as normal tissue.

Various studies have demonstrated the biologically effective dose for SABR to be >100 BED (with alpha/beta ratio of 10) [15,16]. 

A review of retrospective data has shown that tumours of less than and 2 cm have control rates independent of the SABR dose. For tumour sizes of >2 cm, higher control rates were seen (76.2% vs. 60.6%) at 5-year follow-up (*p* = 0.022) with 60 Gy in four fractions (biologically effective dose [BED] = 150 Gy_10_) as compared with 48 Gy in four fractions (BED = 106 Gy_10_) [17].

## 5. Radiotherapy Quality Assurance

Delivering high doses per fraction and motion management remains a very important consideration in SABR delivery due to treatment complexity. Patient specific quality control remains essential for safe delivery. The SABR consortium has played a pivotal role in developing SABR standards within UK. The guidelines are widely adopted and followed within UK in centres that have developed the service. 

A UK-wide audit demonstrated adherence to quality standards with image guidance and tumour motion management for SABR treatment with 74% of SABR treatments being peer reviewed [18]. 

Locally we have audited 130 cone beam CT scans data to calculate intra-fractional motion errors. Our results showed a PTV margin of 0.5 cm was appropriate and comparable to other centres in the UK, accounting for all geometric errors in lung SABR treatment [19].

## 6. Platforms for SABR

Different platforms are available for the delivery of SABR. These include Linac-based, CyberKnife (CK) platform (Robotic arm), Tomotherapy and MRI-cobalt linacs [20,21].

At University Hospitals Birmingham, we have the facility to use both Linac-based and CK platforms. We first treated lung cancer patients using VMAT SABR in June 2013 and with CK in June 2014 [22].

Data was collected retrospectively comparing both platforms showing more T1 tumours treated with VMAT. This was due to our local departmental protocol of excluding tumours less than 2 cm for treatment with CK due to the limits of on treatment localization.

Of the total 241 patients that were treated from June 2013 till February 2017, 153 were treated using VMAT and 88 using CK; 19 patients were excluded from analysis due to lack of follow-up data (18 VMAT, 1 CK). Similar demographics were noted between two cohorts for age, sex and histology although the majority of patients were treated based on radiological diagnosis. 

Local control (LC) was 95.2% for VMAT compared to 93% for CK at median follow-up of 13 months (Range 0.5–45 months). Median OS was 13 months for VMAT compared to 14 months for CK. We saw a higher proportion of disease progression (local or distant) in the VMAT group (25.8% vs. 17.0%). Lung cancer related deaths were equal in both groups (8.9% VMAT vs. 8.0% CK). In our analysis neither platform had any grade 3 or higher toxicities reported. 

## 7. Treating Multiple Lung Tumours with SABR Simultaneously

Multiple lung tumours have been treated with SABR with no reports of significantly increased toxicity [23,24]. There does, however, remain a concern that treating multiple lung lesions will increase the risk of radiation induced lung toxicity.

Tekatli et al. [25] reported 2% Grade ≥3 toxicity, with multivariable analysis showing grade 2 or higher radiation pneumonitis (*n*  =  9) when treating multiple (up to 3) tumours at the same time. Data from 84 patients was reported with 56 patients having bilateral disease. A total lung V35Gy of ≥6.5% (in 2Gy/fraction equivalent) (*p* =  0.007) was found to be the best predictor of toxicity in the study. Median OS was 27.6 months for primary tumours and was not reached for metastatic lesions (*p* = 0.028) with 28 months median follow up.

We have also reviewed the treatment of multiple tumour volumes on the same, rather than on alternate days [26]. We retrospectively reviewed data from our centre for treating multiple lung tumours with SABR simultaneously from April 2016 to May 2017 and have not identified any increase in toxicity with this approach.

Patient demographics showed 2:1 female to male ratio, with 38.9% of patients being ECOG performance status 1. This included bilateral lung cancers, and metastases from colorectal/head and neck and renal cell cancer primaries. 88% of patients had 2 lesions treated with the remaining having 3 lesions. All patients were treated on the same day. Data was collected, and careful consideration was given to the dosimetric analysis particularly Mean Lung dose and lung V20.

The dose fractionations used were 60 Gy in 8#, 55 Gy in 5#, 45 Gy in 5#, 30 Gy in 5#, and 54 Gy in 3#. Mean V20 was 11.6% (range 7.1–23.9%) and Mean Lung Dose (MLD) 8.0 Gy.

This analysis suggested no significant increase in toxicity at the median follow up of 12 months (range 3–20 months). No grade 3 toxicity is reported. Grade 1 and 2 toxicity within 6 weeks, 3 months and long term (9–20 months) reported in Table 2. Local control of treated lesions at 12 months is 75%.

Our experience shows that multiple lung tumours can be safely treated on the same day with no significant increase in toxicity. Prospective data, however, is desirable.

## 8. Toxicity with Lung SABR

Lung SABR has been found to be extremely well tolerated. In the reported literature for the treatment of peripheral tumours Grade 4 toxicities are uncommon and grade 5 rare. Most studies have adequately reported on the toxicities using Common Terminology Criteria for Adverse Events (CTCAE v3), with the most common reported toxicities being breathlessness, pneumonitis, chest wall pain and pneumonia. Fatigue is almost universally reported, although graded mostly at 1 and 2.

Kim et al. [27] reported larger tumour volume to be predictive factor for higher grade radiation pneumonitis. The study reported on 52 primary tumours treated with doses over 100 Gy (BED with α/β value of 10). The risk of grade 2 radiation pneumonitis was reported at 15% with 2.7% risk of grade 3 toxicity. An iGTV (internal GTV) over 4.21 mL [(iGTV) >4.21 mL had a higher pneumonitis rate than with ≤4.21 mL (29.7% vs. 6.1%; *p* = 0.017)] and a PTV of over 14.35 mL was a significant predictive factor for symptomatic radiation pneumonitis. 

Jumeau et al. [28] have reported on chest wall (CW) toxicity (rib fracture and pain). They have suggested that CW V37; a dosimetric parameter adapted to fractionation, may potentially limit CW toxicity after lung SABR. This however, this needs to be tested in prospective studies.

Our experience with toxicity post SABR treatment has not identified any statistically significant difference in pulmonary function tests or quality of life 9 (QoL) (assessed at early i.e., 13–28 and late 30–75 days post SABR treatment) compared to baseline [8].

The low risks of SABR treatment-related toxicity makes it a desirable treatment option with favourable outcomes. Further prospective data particularly for central and ultra-central tumour locations will further guide the management of these tumours.

## 9. Ongoing Research and Future Prospects

Current UK trials HALT [29] and SARON [30] are employing SABR in different systemic settings. HALT trial is a phase II/III study aiming to evaluate if the addition of SBRT to treat limited (≤3) sites of oligoprogressive disease with continuation of current Tyrosine Kinase Inhibitors (TKI) therapy improves progression-free survival outcomes in patients compared with continuation of TKI alone. Transition to full phase III is dependent on feasibility and safety data obtained during the phase II study.

SARON [30] trial aims to assess the addition of SABR to standard chemotherapy in patients with oligometastatic non-small cell lung cancer with randomisation to receive either standard treatment alone (platinum-based doublet chemotherapy) or standard treatment with conventional radiotherapy (RT) and SABR.

SABRTooth [31] and STABLE-MATES [32] trials are comparing SABR directly to surgery in stage 1 lung cancers. The SABRTooth feasibility trial is determining the feasibility and acceptability of conducting a phase III randomised controlled trial comparing SABR with surgery in patients with peripheral stage I non-small cell lung cancer (NSCLC) considered to be at higher risk of complications from surgical resection. STABLE-MATES trial from the United States compares sub-lobar resection to SABR in high risk peripheral tumours. Similarly, VALOR trial [33] is looking (Veterans Affairs study) at lobectomy/segmentectomy compared to SABR in central and peripheral tumours.

The LungTech trial [34] is aiming to investigate the toxicity and safety of treating central lung tumours with SABR and is expected to answer important questions. At present within UK including our centre, primary central lung tumours are not favoured for SABR treatment owing to significant toxicity and treatment related deaths reported in literature. Locally as well we adapt a cautious approach and don’t treat ultra-central and central lung cancers with SABR whilst concrete prospective safety data becomes available.

The difficulty of defining the normal lung tissue dose volume constraints particularly when treating multiple tumours remains an active area of interest. Its dependence on the tumour volume as well as the underlying lung tissue characteristics (i.e., emphysematous changes) and degree of post SABR scarring will remain an important consideration. Although randomised data may not become available for a long period of time, observational data from institutions may steer our experiences and future treatment strategies.

## Figures and Tables

**Table 1:** Comparison of different treatment modalities with pros and cons. [9,10,11,12,13].

Treatment Modality	Radiofrequency Ablation (RFA)	Microwave Thermal Ablation (MTA)	Stereotactic Ablative Radiotherapy (SABR)
	Electromagnetic radio waves with frequencies of less than 1 MHz generating electric field	Part of the electromagnetic spectrum with frequencies between 300 MHz and 300 GHz creating an ellipsoidal microwave field	X rays (6MV photons)
Side effects	Intra/post procedural pain Risk of chest wall burns (subpleural location) Needle-tract seeding Higher risk of rib fractures Pneumothorax risk (11–67%) Haemorrhage/Haemoptysis Infection/abscess Broncho-pleural fistula (rare) Pulmonary artery aneurysms and systemic air embolisms (rare)	Intra/post procedural pain Needle-tract seeding Lower risk of rib fractures Pneumothorax risk (8.5–63%) Broncho-pleural fistula (rare) Haemorrhage/haemoptysis Pleural effusion Infection/abscess	Fatigue Chest wall pain Nausea Dyspnoea Cough/chest infection Pneumonitis Rib fractures Chronic thoracic pain
	Conscious sedation or general anaesthesia	Conscious sedation or general anaesthesia	No conscious sedation or general anaesthesia
	One session	One session	More than one sessions
	Difficult for central lesions	Difficult for central lesions	Technically deliverable, safety data awaited
	Operator dependant	Operator and system dependant	Non-operator dependant
	Size cut off ≤5 cm Preferable size ≤3 cm	Size cut off ≤5 cm Preferable size ≤3 cm	Size cut off ≤5 cm
Location	Peripheral Location dependant e.g., tumours close to scapula, apical etc.)	Peripheral Location dependant e.g., tumours close to scapula, apical etc.)	Peripheral location independent
Treatment time (for a similar-sized treatment area)	12–15 min	2–5 min	Up to 20 min
	More heat sink effect, dependent on electrical permittivity of the tissue	Less heat sink effect	NA
	Smaller ablation zone	Larger more spherical ablation zone	NA
1 year OS	68.2–95%	91%	81–85.7%
3 year OS	36–87.5%	43%	42.7–56%
5 year OS	20.1–27%	-	47%

**Table 2:** CTCAE v3 grade 1–2 acute and long term (LT) toxicity.

Time Scale	Toxicity	*n* = Number of Patients
Acute toxicity within 6 weeks	Fatigue	8
	Chest pain	2
	Skin reaction	1
	Increasing dyspnoea	2
	Loss of appetite	2
	Nausea	1
	Dyspepsia	1
	Cough	4
	Pneumonitis	1
Acute toxicity within 3 months	Fatigue	8
	Chest pain	2
	Skin reaction	1
	Increasing dyspnoea	2
	Loss of appetite	2
	Nausea	1
	Dyspepsia	1
	Cough	5
	Pneumonitis	2
Data from 17/18 pts—one excluded as no acute follow up data		
Long Term (LT) toxicity	Increasing dyspnoea	2
	Chest pain	2
	Cough	1
	Fibrosis/Pneumonitis	2
	Angina symptoms worse	1
Data from 16/18 pts—two excluded as no LT follow up data		
